# Antioxidant therapy for infertile couples: a comprehensive review of the current status and consideration of future prospects

**DOI:** 10.3389/fendo.2024.1503905

**Published:** 2025-01-09

**Authors:** Ramadan Saleh, Hassan Sallam, Mohamad AlaaEldein Elsuity, Sulagna Dutta, Pallav Sengupta, Ahmed Nasr

**Affiliations:** ^1^ Department of Dermatology, Venereology and Andrology, Faculty of Medicine, Sohag University, Sohag, Egypt; ^2^ Ajyal IVF Center, Ajyal Hospital, Sohag, Egypt; ^3^ Department of Obstetrics and Gynaecology, University of Alexandria, Bab Sharqi, Alexandria Governorate, Alexandria, Egypt; ^4^ Alexandria Fertility and IVF Center, Alexandria, Egypt; ^5^ Basic Medical Sciences Department, College of Medicine, Ajman University, Ajman, United Arab Emirates; ^6^ Centre of Medical and Bio-Allied Health Sciences Research, Ajman University, Ajman, United Arab Emirates; ^7^ Department of Biomedical Sciences, College of Medicine, Gulf Medical University, Ajman, United Arab Emirates; ^8^ Department of Obstetrics and Gynaecology, Assiut University, Assiut, Egypt

**Keywords:** antioxidants, female infertility, male infertility, reactive oxygen species, oxidative stress

## Abstract

Oxidative stress (OS) is established as a key factor in the etiology of both male and female infertility, arising from an imbalance between reactive oxygen species (ROS) production and the endogenous antioxidant (AOX) defenses. In men, OS adversely affects sperm function by inducing DNA damage, reducing motility, significantly impairing sperm vitality through plasma membrane peroxidation and loss of membrane integrity, and ultimately compromising overall sperm quality. In women, OS is implicated in various reproductive disorders, including polycystic ovary syndrome, endometriosis, and premature ovarian failure, leading to diminished oocyte quality, disrupted folliculogenesis, and poorer reproductive outcomes. Antioxidant therapy represents a promising intervention to mitigate the harmful effects of ROS on reproductive health in additions to its easy accessibility, safety, and low cost. Despite several findings suggesting improvements in fertility potential with AOX therapy, the data remains inconclusive regarding optimal dosage and combination, duration of treatment, and the specific patient populations most likely to benefit. In this review, we discuss the role of AOXs in the management of infertile couples, focusing on their biological mechanisms, potential adverse effects, therapeutic efficacy, and clinical applications in improving reproductive outcomes in both natural conception and medically assisted reproduction. Additionally, we highlight the current practice patterns and recommendations for AOX supplementation during the course of infertility treatment. Further, we provide an overview on the limitations of the current research on the topic and insights for future studies to establish standardized AOX regimens and to assess their long-term impact on key outcomes such as live birth rates and miscarriage rates.

## Introduction

The term oxidative stress (OS) applies when increased amounts of reactive oxygen species (ROS) surpass the antioxidant (AOX) capacity that naturally offers the protective balance (1). While OS plays a major role in the pathogenesis of male infertility (2–4), it is equally important to recognize the critical physiological roles of ROS in normal reproductive functions. ROS are indispensable for processes such as sperm capacitation, hyperactivation, and the acrosome reaction, which are essential for successful fertilization (5, 6). However, an imbalance favoring excessive ROS production can lead to oxidative damage, negatively affecting male fertility potential and reproductive outcomes (7, 8). Also, OS has been linked with many female infertility conditions such as polycystic ovary syndrome (PCOS) (9, 10), endometriosis (11–13), premature ovarian failure (POF) (14), and recurrent miscarriages (RM) (15).

Hence, AOX therapy to enhance fertility by protecting against the harmful effects of ROS is theoretically feasible. AOXs are not regulated as pharmaceutical drugs, thus allowing their utilization without prescription. Indeed, AOXs are extensively consumed due to their easy availability through different retail outlets (16). Also, the relatively low cost and good safety profile of AOX products are appealing to both healthcare providers and infertile couples. However, a significant diversity exists among practitioners in terms of prescribing AOXs for the purpose of infertility treatment (17).

In this review, we comprehensively discussed the types, mechanisms of action and safety profile of AOXs that are commonly prescribed in infertility clinics. We also highlight the attitudes and practice patterns of clinicians towards AOX prescription during the course of infertility treatment, and compare them with current recommendations by professional societies. Additionally, we discuss the impact of AOX therapy on fertility outcomes in men and women under natural and assisted reproduction conditions. Furthermore, we highlight limitations of the current research on utility of AOXs for treatment of infertile couples and the need for well-designed studies that can bridge the gap between research and practice in this regard.

## Types, natural sources and mechanism of action of antioxidants

An AOX is a natural substance that provides protection to the living cell against oxidative damage and deleterious effects of ROS (18). Under normal circumstances, there is a fine balance that enables AOXs to neutralize excess ROS, and maintain a small amount of ROS necessary for normal cell functions. Thus, when ROS production increases to harmful levels, AOXs play a key role in neutralizing them. In the living cell, the AOX defense system is generally classified into enzymatic and non-enzymatic elements.

Enzymatic AOXs include glutathione (GSH) peroxidase, glutathione reductase, catalase (CAT) and superoxide dismutase (SOD) (19). Nonenzymatic AOXs provide protection against ROS by binding to enzymatic AOXs and ions that helps subsequent suppression of enzymes involved in the oxidation process (16). They function by scavenging free radicals, stabilizing enzymatic AOXs, and chelating metal ions, thereby mitigating OS. Prominent non-enzymatic AOXs include vitamins such as Vitamin C and Vitamin E (17, 20, 21), glutathione (22, 23), coenzyme Q10 (24–27), carotenoids (e.g., beta-carotene, lycopene) (28), polyphenols (29), and trace elements like selenium and zinc (24, 30). AOXs are available in the market as individual or combined products. Generally, AOXs utilized in clinical practice are categorized as substances with direct AOX actions or substances with AOX properties (16). **Table 1** summarizes the types, natural dietary sources and the mechanism(s) of action of AOXs.

**Table 1:** Types, dietary sources and mechanism of action of antioxidants.

Supplement	Mechanism of action	Dietary source
Supplements with direct antioxidant action		
Carnitines	• Carnitine is found in the epididymis, seminal plasma and in spermatozoa, • Assists sperm metabolism by positively affecting sperm motility and maturation	Carnitines are present in meat, fish, poultry and dairy.
Carotenoids	• β carotene is a provitamin A, which can directly scavenge ROS, • β carotene protects lipid membranes from peroxidation, • β carotene improves sperm motility and morphology.	Carotenoids are present in leafy green vegetables, fruits, and some vegetable oils.
Vitamin C	• Diminishes DNA damage by scavenging free radicals and decreasing formation of lipid hydroperoxides.	Vitamin C is present in citrus fruits and vegetables.
Vitamin E	• Vitamin E is the first defense against oxidant-induced membrane injury.	Vitamin E is present in vegetable oils (like wheat germ, sunflower, and safflower oils), nuts (such as peanuts, hazelnuts, and almonds) and seeds (like sunflower seeds).
Coenzyme Q10 (CoQ10) (ubiquinol)	• CoQ10 is a fat-soluble AOX synthesized endogenously. • An essential component of the mitochondrial energy metabolism.	CoQ10 is present in meat, fish, nuts and some oils.
Arginine/L-arginine	• Arginine is required for normal spermatogenesis. • Arginine directly protects against oxidative damage, • Arginine plays a role in the inflammatory response.	Arginine is present in meat products, dairy, nuts and seeds.
Cysteine and N-acetyl cysteine (NAC)	• Cysteine plays an important role in glutathione synthesis. • NAC is a precursor of the amino acid cysteine and a direct scavenger of ROS.	Cysteine and NAC are present in meat, fish, seafood, chicken or turkey, eggs, whole-grain products such as breads and cereals, broccoli, onions, and legumes.
Folic acid (folate or vitamin B9)	• Important for the synthesis of DNA, transfer RNA and the amino acids cysteine and methionine. • Scavenges oxidizing free radicals and inhibit lipid peroxidation.	Folate is present in green-leafy vegetables, liver, bread, yeast and fruits.
Selenium	• Selenium is involved in the cellular AOX defense by increasing the activity of the AOX enzyme glutathione peroxidase. • Selenium is essential for normal spermatogenesis.	Selenium is available in fish, meat products, and diary.
Zinc	• Zinc is involved in DNA transcription and protein synthesis, • Zinc has extensive antioxidants properties. • Zinc has an important role in testes development and sperm functions.	Zinc is available in meat products, wheat and seeds.
Supplements with antioxidant properties		
Vitamin B (complex)	• Vitamin B is a water-soluble vitamin and consists of several precursor and coenzymes such as thiamine (B1), riboflavin (B2) and cobalamin (B12). • Vitamin B plays an important role in lowering plasma levels of homocysteine, thus reducing the pro-oxidant effects of the latter.	Vitamin B is mainly found in meat products, other examples of food sources are beans, potatoes, bananas and mushrooms.
Vitamin D	• Vitamin D is a fat-soluble vitamin, with the natural main source being dermal synthesis (sun light). The active form of vitamin D is 1,25-dihydroxyvitamin D. • Vitamin D3 has AOX properties mainly by inducing the antioxidant protein superoxide dismutase	Fish liver oil, oily fish, salmon, sardines, red meat, liver, egg yolk, and dairy products.
Myo-inositol	• Inositol is a polyalcohol, naturally occurring as nine stereoisomers including myo-inositol (MYO). • Myo-inositol, is considered as a “pseudovitamin”. • Myo-inositol is produced by Sertoli cells in response to follicle-stimulating hormone (FSH). • Inositol plays an important role in cell membrane formation and lipid synthesis. • The seminiferous tubules contain the highest concentration of myo-inositol • Myo-inositol is directly involved in regulation of motility, capacitation and acrosome reaction. • Myo-inositol acts by increasing endogenous AOX enzymes and directly enhancing the mitochondria membrane potential.	
Polyunsaturated fatty acids (PUFAs)	• Omega 3 might have a free radical-scavenging potential. • PUFAs have a prooxidant rather than a direct antioxidant effects. • PUFAs increase the plasma fluidity of the sperm membrane. This fluidity makes the sperm vulnerable to damage by ROS and lipid peroxidation.	The main sources of PUFAs are vegetable and fish oils. Omega-9 is synthesized by animals, but omegas-3 and -6 need to be supplemented in the diet.
Resveratrol	• Resveratrol is a natural phytoalexin with antioxidant properties. • Resveratrol enhances AOX defenses and improves sperm motility.	Resveratrol is naturally found in grapes, berries, and several nuts.
Lycopene	• Lycopene is an AOX that plays many roles including cell growth regulation, gap junction communication, gene expression modulation, immune response, and protection of lipid peroxidation.	Lycopene belongs to the family of carotenoids found in many bright fruits and vegetables (e.g., tomato) that includes alpha-tocopherol, alpha-carotene, beta-cryptoxanthin, beta-carotene, and lutein, and is the most effective AOX among the carotenoid family.

AOX, Antioxidant; CoQ10, Coenzyme Q10; NAC, N-acetyl cysteine; ROS, Reactive oxygen species.

## Safety profile of antioxidants

AOXs are generally well tolerated and have a good safety profile. However, the fact that AOXs are largely available over-the-counter with a reasonable price increases the risk that some patients can overmedicate with AOXs leading to detrimental effects. It has been suggested that administration of large amounts or improper combinations of AOXs may lead to shift of the redox balance towards a ‘reductive stress’ status (31). The term ‘antioxidant paradox’ highlights the potential negative impact of over-utilization of AOX supplementation (32, 33). Recent reports indicate that the intake of excess AOXs may even cause impairment of male fertility potential due to reductive stress (34, 35). However, there is no clear understanding of the exact mechanism of action of reductive stress on male reproduction, and further research is required. Additionally, high doses of AOXs may be associated with some adverse effects. Furthermore, it is imperative to consider the role of excipients or fillers in commercially available supplements. For instance, titanium dioxide, previously regarded as a safe additive, has recently been implicated in genotoxicity, raising concerns about its safety. These findings underscore the need for a judicious and evidence-based approach in the formulation and administration of AOX supplements to ensure both their safety and therapeutic efficacy (36, 37). A summary of potential adverse effects of AOXs frequently used in the treatment of infertile couples is provided in **Table 2** (38–54).

**Table 2:** Potential adverse effects of antioxidants.

Supplement	Adverse effects
Carnitines	• Administration of daily doses of carnitines above 3 g daily can give gastrointestinal side effects and malodorous effects (38, 39).
Carotenoids	• Excess intake of vitamin A can lead to toxicity (hypervitaminosis A), which is manifested by nausea, irritability, reduced appetite, vomiting, blurry vision, headaches, hair loss, muscle pain, papilledema, hemorrhage, weakness, drowsiness and altered mental status (40). • Excess intake of provitamins such as carotenoids may lead to carotenaemia (yellow-tinged skin) (41).
Vitamin C	• In some people, administration of vitamin C may lead to nausea, vomiting, headache, flushing, fatigue and joint pain (42).
Vitamin E	• Excess vitamin E may increase bleeding risk (43).
Coenzyme Q10 (CoQ10) (ubiquinol)	• CoQ10 may cause mild gastrointestinal symptoms (44).
Arginine/L-arginine	• No significant adverse events have been observed. • Arginine is contraindicated for patients a history of genital or oral herpes, asthma or cancer (45).
Cysteine and N-acetyl cysteine (NAC)	• Up to 8000 mg daily oral NAC does not cause significant adverse events (46).
Folic acid (folate or vitamin B9)	• Folic acid doses of 5 mg/day and over can cause abdominal cramps, diarrhea and rash. • Higher doses of folic acid can cause altered sleep patterns, irritability, confusion, exacerbation of seizures and nausea (47).
Selenium	• Excess intake of selenium may lead to a garlic odor in the breath and a metallic taste in the mouth. • Prolonged intake of high selenium may lead to gastrointestinal symptoms, fatigue, hair loss, joint pain and nail problems (48).
Zinc	• High intake of zinc may lead to loss of appetite, nausea, vomiting, abdominal pain and diarrhea (49).
Vitamin B (complex)	• High doses of vitamin B may lead to gastrointestinal symptoms, skin flushing, skin rash or liver impairment (50).
Vitamin D	• High intake of vitamin D may lead to loss of appetite, nausea, vomiting, constipation, dry mouth, arrythmia, hypotension and weakness (51).
Myo-inositol	• High doses of inositol may cause nausea, abdominal pain, diarrhea, headache, dizziness and difficulty sleeping (52).
Polyunsaturated fatty acids (PUFAs)	• Intake of PUFAs may cause fishy taste in the mouth and gastrointestinal symptoms such as vomiting, diarrhea and abdominal pain (53).
Resveratrol	• Resveratrol may cause some reversible gastrointestinal side effects (54).
Lycopene	• High doses may result in gastrointestinal disturbances such as nausea, diarrhea, and potential skin discoloration (90)

Doses given are for adult male. AOX, antioxidant; CoQ10, Coenzyme Q10; NAC, N-acetyl cysteine; PUFAs, Polyunsaturated fatty acids.

## Antioxidant therapy of male infertility

**Figure 1:**
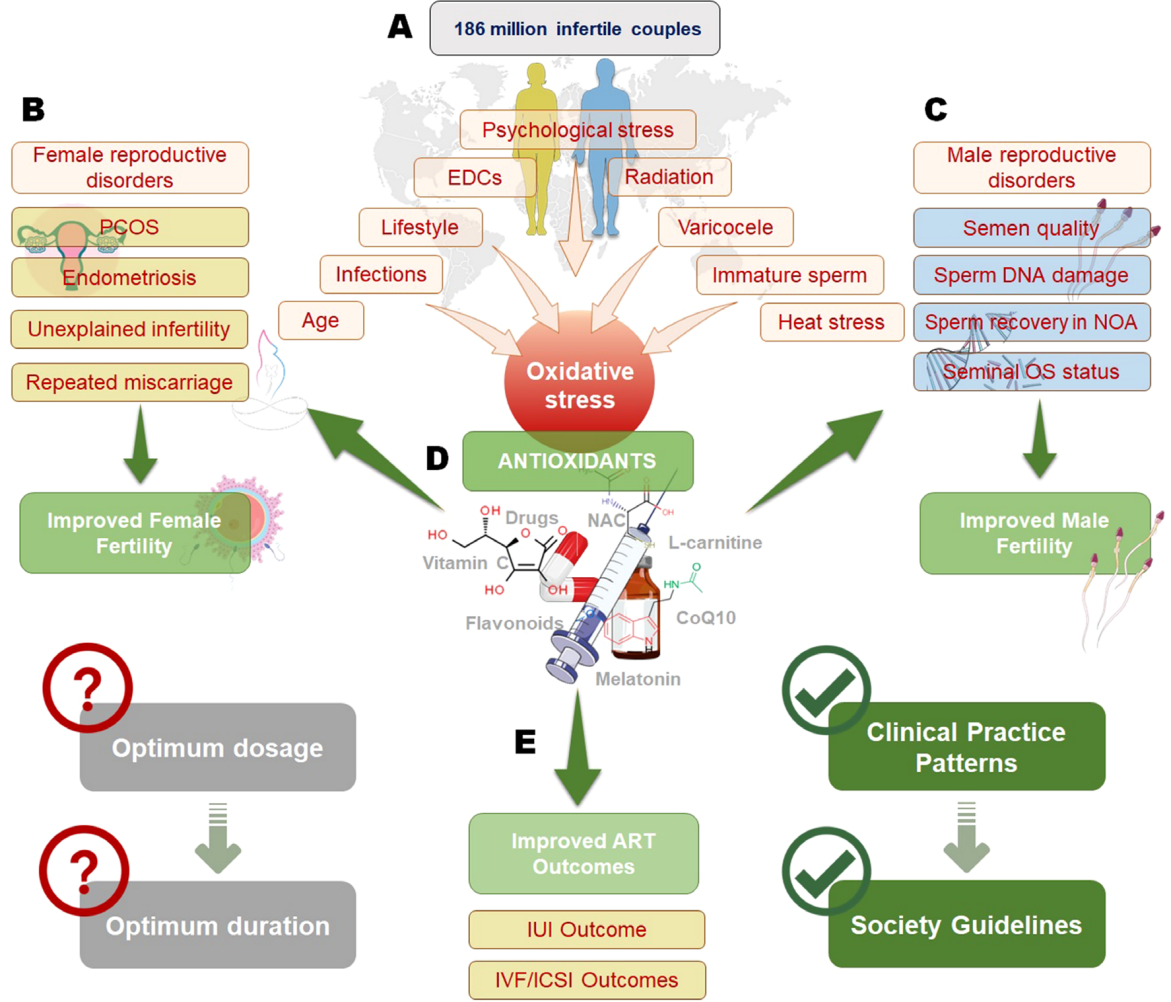
Impact of oxidative stress on infertility and the role of antioxidants in improving reproductive outcomes. Oxidative stress, influenced by factors such as psychological stress, endocrine-disrupting chemicals (EDCs), radiation, varicocele, infections, age, and lifestyle, affects both male and female fertility **(A)**. In females **(B)**, oxidative stress contributes to conditions like polycystic ovary syndrome (PCOS), endometriosis, unexplained infertility, and recurrent miscarriage, while in males **(C)**, it impacts semen quality, sperm DNA integrity, non-obstructive azoospermia (NOA), and seminal oxidative stress status. Antioxidant therapy, including vitamins, flavonoids, N-acetylcysteine (NAC), L-carnitine, and coenzyme Q10 (CoQ10), plays a crucial role in mitigating oxidative stress and improving both male and female fertility **(D)**. However, determining the optimum dosage and duration of antioxidant therapy remains a challenge. Antioxidants have also been shown to enhance outcomes in assisted reproductive technologies (ART), such as intrauterine insemination (IUI) and *in vitro* fertilization/intracytoplasmic sperm injection (IVF/ICSI), contributing to the development of clinical guidelines for infertility management **(E)**.

Increased public awareness of the positive impact of nutrition on different body functions highlights the significance of dietary modification as an important strategy to enhance fertility potential (**Figure 1**). A healthy diet or an extra dietary intake of AOXs has been correlated with high sperm quality in healthy individuals (55–58).

### Rationale of antioxidant therapy of infertile men: role of oxidative stress

In the male genital tract, seminal ROS are produced by sperm, white blood cells, and as by- products during various metabolic processes. Small amounts of ROS are required for normal sperm functions such as sperm capacitation and acrosome reaction (2, 59). Excess ROS production may occur under different circumstances such as smoking (60), leukocytospermia (61), and varicocele (62). Male Oxidative Stress Infertility (MOSI) defines a group of infertile men with abnormal semen analysis results and excess ROS in their semen (63). This category of patients was previously classified as having idiopathic male infertility (IMI). The high prevalence of IMI highlights the significance of MOSI diagnosis during infertility workup. OS significantly impairs male fertility through disruption of sperm capacitation, induction of DNA damage such as sperm DNA fragmentation (SDF), reduced motility, and diminished sperm membrane fluidity. These detrimental effects are further exacerbated by a marked decline in sperm vitality, resulting from plasma membrane peroxidation and loss of membrane integrity. Together, these factors compromise overall sperm quality and hinder the fertilization process (64, 65).

AOX supplementation has demonstrated significant potential in managing MOSI, as highlighted by a systematic review encompassing 97 clinical trials. These studies revealed the beneficial effects of AOX supplementation in men with (a) abnormal semen quality, (b) varicocele, and (c) idiopathic or unexplained infertility (66). However, the efficacy of AOX therapy relies heavily on the accurate identification of OS through reliable diagnostic testing. Assessing OS biomarkers, including ROS levels, total antioxidant capacity (TAC), lipid peroxidation products and DNA fragmentation, is critical for detecting redox imbalances. Advanced diagnostic techniques, such as chemiluminescence, ELISA, and sperm-specific OS tests, provide precise evaluations of OS in semen (67–69). By employing this targeted diagnostic approach, AOX therapy can be personalized for individuals with confirmed OS, avoiding the risks associated with empirical or excessive supplementation, such as reductive stress (7). Integrating OS testing into clinical protocols ensures optimized therapeutic outcomes, personalized interventions, and minimizes potential adverse effects related to inappropriate AOX use.

The impact of different AOX supplementations to improve fertility outcomes in infertile men has been the subject of extensive research in the last few decades. Additionally, the growing interest in AOX therapy for male infertility has led to the development of variety of AOX combinations. In the male, AOXs may enhance fertility through preserving sperm DNA integrity and mitochondrial transport (70). The AOXs that are commonly used in published studies include vitamin E, vitamin C, carotenoids, carnitine, cysteine, coenzyme Q10 (CoQ10), selenium, zinc, and folic acid (16, 24–26, 30, 71–90).

### Potential indications of antioxidant supplementation to enhance male fertility

Beyond MOSI, several clinical conditions and lifestyle factors warrant consideration for AOX therapy in male infertility treatment. Chronic diseases, specifically diabetes mellitus and obesity, have been associated with increased seminal OS and subsequent fertility impairment (91–93). In diabetic men, hyperglycemia-induced OS leads to increased SDF and reduced sperm motility, making them potential candidates for AOX supplementation (93).

Environmental and occupational exposures represent another significant indication for AOX therapy. Men exposed to heavy metals, pesticides, or electromagnetic radiation often exhibit elevated OS markers in seminal plasma (94). Cancer treatments significantly elevate OS through multiple mechanisms: chemotherapeutic agents, particularly alkylating agents and platinum compounds, generate excessive ROS directly damaging sperm DNA and membranes (87, 88), while radiation therapy induces both acute and chronic OS states in testicular tissue, leading to prolonged spermatogenic dysfunction (95).

Recently, attention has focused on men recovering from Coronavirus disease-19 (COVID-19) infection as potential candidates for AOX therapy. It has been suggested that COVID-19 infection may impact male reproductive function through OS-mediated mechanisms (96). While more research is needed, AOX supplementation might play a role in reproductive recovery post-infection (97).

### Antioxidant therapy of infertile men: recommendations of professional societies

To date, results of studies on AOX therapy of infertile men are controversial, and no firm conclusion could be reached. Therefore, the guidelines of professional societies do not recommend the routine use AOXs in treatment of male infertility, mainly due to the heterogeneity of the available data. This should not be construed as a contraindication to the use of AOXs; rather, it underscores the necessity for more robust, high-quality, and standardized evidence to substantiate their routine application in clinical practice. The latest guidelines by the European Association of Urology state that “no conclusive data are available regarding the beneficial treatment with AOXs in men with idiopathic infertility, although they may improve semen parameters” (98). Similarly, the current American Urological Association and American Society for Reproductive Medicine guidelines question the benefits of AOXs in treating male infertility, and indicate lack of data to recommend the use of specific agents for this purpose (Moderate Recommendation; Evidence Level: Grade B)” (99).

### Antioxidant therapy of infertile men: impact of on fertility outcomes

#### Basic semen parameters

Results emerging from the review by Agarwal et al. (2022) indicated a significant increase of basic sperm parameters including sperm concentration, total motility, progressive motility and normal forms following AOX therapy versus placebo or no treatment (100). However, these results should be interpreted with caution due to high heterogeneity that was observed among the studies included in this meta-analysis. Data of another meta-analysis also indicate better semen quality in infertile men who received AOX supplementation after varicocelectomy (101). A previous systematic review also reported that administration of AOXs such as vitamin E, vitamin C, N-acetyl cysteine (NAC), carnitines, CoQ10, lycopene, selenium, and zinc, resulted in significant improvement of sperm concentration, motility, and morphology (102). Further, a recent meta-analysis examined the impact of supplementation with L-Carnitine/ALC or NAC on semen quality and had the same conclusion (81). Further, *in vitro* glycine supplementation has been found to improve the quality of cryopreserved sperms in an animal experiment (103).

According to data from the Cochrane review, administration of carnitines and combined AOX formulations had positive effect of sperm motility, and supplementation with polyunsaturated fats and zinc resulted in increase of sperm concentration (16). Zinc supplementation plays a pivotal role in enhancing spermatogenesis through its diverse functions in male reproductive physiology. It is indispensable for spermatogenesis, as it supports the functionality of Sertoli cells and maintains the structural integrity of the seminiferous epithelium, which is essential for effective sperm production. Zinc also serves as a cofactor for critical enzymes involved in DNA synthesis, repair, and cellular division, thereby facilitating the proliferation and differentiation of spermatogenic cells. Additionally, its antioxidant properties help counteract OS, shielding germ cells from damage induced by ROS. Moreover, zinc regulates testosterone secretion, a hormone essential for sustaining optimal spermatogenic activity and promoting healthy sperm concentration. Despite abundance of studies reporting positive outcome of AOX therapy on basic sperm parameters, it is difficult to get a firm conclusion due to the high heterogeneity among published studies.

#### Sperm recovery in patients with non-obstructive azoospermia

A study investigated the outcome of three-month supplementation with multivitamins, micronutrients and CoQ10 in infertile men with non-obstructive azoospermia (n=24) as compared to no treatment (n=11) (104). Histological evaluation of testicular biopsies of azoospermic patients showed maturation arrest mostly at spermatid level. At monthly follow up visits, the semen analysis of the treated group showed recovery of motile sperm in the ejaculate at concentration of 4.0 million/ml, while the untreated groups remained unchanged. Authors of the latter study reported further increase of sperm density on subsequent visits, and 2 pregnancies within 3 months. However, these results should be taken with caution due to poor study design and high drop-out ratio (105). More importantly, these results were not replicated in further studies.

#### Sperm DNA fragmentation

Folic acid supplementation resulted in lowering the levels of SDF in carriers of *MTHFR* gene TT polymorphism (106). However, a meta-analysis found no significant difference in SDF levels in infertile men following AOX supplementation as compared to placebo or no treatment (100). Of note, only small number of sudies investigated this outcome with heterogenity in inclusion criteria among study populations, and differences in AOX combinations and SDF assays used. Future studies are warranted to examine the effacacy of AOX therapy in men with high SDF.

#### Seminal oxidative stress indices

Few studies demonstrated improvement in OS as determined by reduction of levels of malonaldehyde (MDA), a lipid peroxidation byproduct following therapy with Vitamin C, vitamin E, beta-carotene, zinc, selenium, and NAC when used alone or in combination (107–111). A recent meta-analysis indicates a significant increase in seminal AOX and reduction of MDA following AOX therapy although significant inter-study heterogeneity was detected (112). MDA, a byproduct of lipid peroxidation, serves as a key biomarker of OS and exhibits cytotoxic effects on spermatozoa. Elevated MDA levels compromise the structural integrity and fluidity of the sperm membrane, resulting in diminished motility and reduced viability (113, 114). Furthermore, MDA can directly interact with DNA, causing strand breaks and base modifications, thereby adversely affecting sperm DNA integrity and reducing fertilization potential (115, 116). Consequently, the observed reduction in MDA levels following AOX therapy highlights its therapeutic significance in alleviating OS and improving outcomes in OS-related male infertility.

#### Clinical pregnancy rate and live-birth rate

Recent years have wetnessed a growing interest in the study of the impact of AOX therapy on clinical pregnancy rate (CPR) and live-birth rate (LBR). In a Cochrane review investigated AOX supplementation protocols for male subfertility, focusing on active substances including carnitines, coenzyme Q10, selenium, zinc, vitamins C and E, and carotenoids. Dosages varied according to the specific AOX, with some studies incorporating combinations of more than three AOXs. The duration of supplementation ranged from three to nine months, with outcomes evaluated at defined intervals of three, six, or nine months. The latter Cochrane review reported that AOX supplementation in infertile men resulted in a significant increase of CPR (odds ratio (OR) 2.97, 95% confidence interval (CI) 1.91-4.63) although the level of evidence was found to be low (16). A similar finding was reported by a recent meta-analysis where AOX therapy of infertile men was associated increased spontaneous CPR (OR 1.97, 95% CI: 1.28, 3.04; p<0.01) (100). In a third meta-analysis, CPR was significantly higher in infertile men with IMI who received combined L-carnitine and acetyl L-carnitine (ALC) supplementation (OR=3.76, p=0.002). The study evaluated the efficacy of combined L-carnitine and ALC supplementation in men diagnosed with idiopathic oligoasthenoteratozoospermia (iOAT). The treatment protocols consisted of LC administered at a dose of 2 g/day and LAC at 1 g/day, with intervention durations spanning 12 to 24 weeks across the included trials. This combination therapy demonstrated potential to improve key semen parameters, particularly forward motility and total motile sperm counts, while also contributing to enhanced pregnancy rates in affected patients (73).

Older reviews have also reported higher CPR in infertile men treated with AOXs whether couples were trying to oncieve naturally or through one of the medically assisted reproduction (MAR) procedures (102, 117, 118). However, a study did not find better pregnancy rates in infertile couples in whom the male partner received CoQ10 supplementation, although sperm parameters were found better (26, 27, 119).

On the other hand, the results of systematic reviews by Cochrane (16) and Agarwal et al. (2022) were unable to show improvement of LBR despite higher pregnancy rates possibly due to scarcity of data available on this outcome (100). This may be attributed to methodological difficulties that preclude performing this kind of studies as they require longer durations of follow up.

#### Miscarriage rate

According to the systematic reviews by Cochrane (16) and Agarwal et al. (2022) (100), AOX therapy of infertile men was not correlated with miscarriage rates mainly due to small number of studies addressing this parameter.

### Dose and duration of antioxidant therapy in male infertility

To date, there is no clear guidance as to the optimum dose or duration of AOX therapy for infertile men. The duration reported by different studies examining the effects of AOX therapy in male infertility is variable ranging from 1 month (120) to 12 months (78). A minimum of 26 weeks is recommended by the US Food and Drug Administration to evaluate the therapeutic potential of an intervention for male factor infertility (121). Based on this recommendation, it is assumed that a period of 6.5 months is needed for AOX therapy in infertile men. For instance, L-carnitine, L-acetyl carnitine, NAC, and vitamin E have been reported to require a duration of 6-12 months (52, 56, 57, 62, 63). The dose of AOX supplementation is also variable and is mainly decided by the manufacturer and according to the experience of the individual practitioner (66). A summary of dose schedules of AOXs frequently used in the treatment of infertile men is provided in **Table 3** (24–26, 30, 73, 74, 76–89).

**Table 3:** Dose schedule of antioxidants commonly used in treatment of male infertility.

Supplement	Dose schedule^*^
Supplements with direct AOX action	
Carnitines	• L-carnitine 2 g daily + L-acetyl carnitine 1g daily for 3-6 months (73)
	•
Vitamin C	• 500 mg daily for 3 months (76).
Vitamin E	• 600 mg daily for 3-12 months (77, 78).
Coenzyme Q10 (CoQ10) (ubiquinol)	• 200-400 mg daily for 3 months (24–26).
Arginine/L-arginine	• Arginine-HCL 500 mg daily for 3 months (79).
Cysteine and N-acetyl cysteine (NAC)	• NAC 600 mg daily for 3-6 months (80, 81).
Folic acid (folate or vitamin B9)	• Folic acid 5 mg daily for 6 months (82)
Selenium	• 200 µg daily for 3 months (24, 80)
Zinc	• 66 mg daily for 3 months (30)
Supplements with AOX properties	
Vitamin B (complex)	• Vitamin B12 25 µg daily for 4 months (83). Methylcobalamin 1500-6000 µg daily for 3-6 months (83).
Vitamin D	• 2500 IU daily for 6 months (84), 4000 IU daily for 3 months (85), or 60000 IU weekly for 6 months (86).
Myo-inositol	• 4 gm daily for 3 months (87).
Polyunsaturated fatty acids (PUFAs)	• eicosapentaenoic (EPA) and docosahexaenoic acids (DHA) 1.84 gm daily for 8 months (88).
Resveratrol	• 150 mg twice daily for 3 months (89)
Lycopene	• 4 to 8 mg daily, for 3 to 6 months (74)

^*^Doses given are for adult male. AOX, antioxidant; CoQ10, Coenzyme Q10; NAC, N-acetyl cysteine; PUFAs, Polyunsaturated fatty acids.

### Practitioners’ attitude towards antioxidant therapy of male infertility

Reproductive specialists continue to prescribe AOXs in their daily practice of male infertility treatment despite lack of clear guidelines (102). The results of a global survey including 1,327 reproductive physicians indicated that 43.7% of the survey participants prescribe AOXs to all patients and another 41.9% prescribe AOXs for selected categories of patients (66). Interestingly, 80% of the clinicians indicated that they prescribe AOXs for treatment of infertility without testing for seminal OS. The results of the survey showed lack of consensus among practitioners regarding the desired treatment outcome. While 18.8% of clinicians suggested basic semen parameters as their primary outcome measure, an equal number selected LBR, and others selected either SDF or CPR. Additionally, a significant diversity was observed among practitioners regarding the AOXs they prescribe with 36 different AOXs being listed. It may be appropriate to use a combination of AOXs that act by different mechanisms to protect spermatozoa against oxidative damage (66). However, no clear guideline is currently available to recommend the use of specific AOX or AOX combinations for male infertility treatment.

## Antioxidant therapy of female infertility

### Rationale of antioxidant therapy of role of female infertility: role of oxidative stress

Although the evaluation of OS in male reproduction can be easily done by examining the semen, the same evaluation is more complicated in women as OS indices in the serum do not accurately reflect the situation in the female reproductive system. Accordingly, more invasive procedures are needed such as examining the follicular fluid obtained during *in vitro* fertilization (IVF) or intracytoplasmic sperm injection (ICSI) or examining the peritoneal fluid obtained by laparoscopy. Consequently, much of the information on OS in female reproduction has been obtained from animal studies. Despite this limitation, OS was shown to be involved in many aspects of female reproduction (122–124). ROS in the ovaries is mainly produced in the macrophages, leukocytes, and cytokines present in the follicular fluid. On the other hand, the ovary contains two types of the AOX enzymes; SOD: copper-zinc SOD (Cu/Zn SOD), mainly present in the Graafian follicle and manganese SOD (MnSOD) concentrated mainly in the corpus luteum (124). ROS are involved in many physiological functions of the ovary such as steroidogenesis, oocyte maturation, ovulation, formation of blastocysts, implantation, luteolysis and maintenance of the luteal phase (124, 125). More specifically, in small doses, they are involved in folliculogenesis, dominant follicle selection and corpus luteum formation in part by regulating angiogenesis (126). In addition, they play a role in dominant follicle selection, as regressing follicles undergo a process of apoptosis which is promoted by ROS. This delicate balance is an interplay between ROS production and the up-regulation of the AOX pathways CAT and GSH which opposes the apoptotic process (124).

ROS are also involved in ovulation, follicular rupture and corpus luteum formation. Besides cytokines, and vascular endothelial growth factor (VEGF), the local production of prostaglandin F2α (PG2 α) leading to vascular ischemia at the site of follicular rupture (i.e. ovulation) stimulates the production of the ROS (122). On the other hand, following ovulation, the increased activity of Cu/Zn-SOD during the early and mid-luteal phase supports corpus luteum function while its diminished activity during the regression phase is associated with luteolysis and cessation of corpus luteum function, ushering menstruation (127).

OS, resulting from increased ROS production or diminished AOX capacity, can negatively impact female reproductive processes. It can damage oocytes, impair follicular development, reduce endometrial receptivity, and disrupt early embryo development, potentially leading to fertilization failure, and implantation issues. Additionally, OS can cause hormonal imbalances that further compromise fertility, indicating the role of ROS-AOX balance in maintaining reproductive health (128). OS was shown to be associated to a varying extend with many reproductive diseases such as the PCOS (9), endometriosis (11–13), POF (129), unexplained infertility (122) and even RM (15). Consequently, AOXs are used with varying degrees of success in the treatment of these conditions (**Figure 1**).

### Antioxidant therapy of polycystic ovary syndrome

The PCOS is the most common female reproductive disorders and affects 5% to 10% of US women (130). The updated 2018 diagnostic criteria for PCOS require the presence of at least two of the following three features: (1) oligo/anovulation, (2) clinical and/or biochemical hyperandrogenism, and (3) PCO identified by the presence of ≥20 antral follicles per ovary on transvaginal ultrasound, with or without increased ovarian volume. These criteria are more stringent than the previous Rotterdam criteria, aiming to identify women with higher metabolic risks associated with PCOS (131).

About two thirds of PCOS patients suffer from insulin resistance often leading to the development of the metabolic syndrome (130). Although many theories have been proposed to explain the pathogenesis of PCOS, recent studies have increasingly linked OS to various aspects of the condition (124, 132). A study demonstrated that hyperglycemia induces excess ROS production from mononuclear cells, which subsequently triggers the release of tumor necrosis factor-α (TNF-α) (105). The resulting inflammatory cascade of events exacerbates OS and insulin resistance. This, in turn, contributes to the formation of multiple ovarian cysts, chronic anovulation, and infertility (133, 134). Another study showed that elevated insulin levels promote the proliferation of thecal cells and increase the secretion of androgens, including luteinizing hormone (LH), while also upregulating the expression of cytochrome P450 and insulin growth factor-1 (IGF-1) receptors (107). The activity of cytochrome P450 is known to generate ROS, perpetuating a vicious cycle of OS. Additionally, plasma levels of L-carnitine were found to be significantly lower in obese PCOS patients compared to their non-obese counterparts (135). Despite these findings, it remains unclear whether OS is a primary factor in the development of PCOS or a secondary consequence of hyperglycemia, insulin resistance, and associated cardiovascular and necrotic complications in these women (124, 132).

Based on these and other findings, AOXs were proposed as a treatment of PCOS either on their own, as a combination or as adjuvants to other agents for ovulation induction in the infertile PCOS patients. For example, Sahelpour et al. (2019) treated 80 PCOS patients with L-carnitine (3g/day) for 3 months (109). The authors found that insulin sensitivity improved significantly and that the serum levels of low-density lipoprotein (LDL) decreased. Additionally, there was a decrease in the body mass index (BMI) and the patients reported that their menstrual cycles became more regular and that their hirsutism diminished (136). In a randomized controlled trial (RCT), 60 overweight PCOS patients were treated with 250 mg of carnitine supplements for 12 weeks, while the control group received a placebo (110). They found that carnitine administration was associated with a significant reduction in BMI, and waist and hip circumferences compared to placebo. They also found a significant reduction in fasting plasma glucose, serum insulin levels, homoeostasis model of assessment-insulin resistance and dehydroepiandrosterone sulphate (DHEAS) in the treated patients. However, in this latter study, carnitine administration had no effect on lipid profiles or free testosterone. NAC is another popular agent used for the treatment of infertile PCOS patients with encouraging results (137). However, a meta-analysis reported that the use of NAC showed no significant improvement in pregnancy rate, serum LH level, fasting insulin, and LH/follicle-stimulating hormone (FSH) ratio despite that the BMI and total testosterone were significantly reduced (138).

Zinc and selenium were also used in the treatment of PCOS based on the fact that serum zinc and selenium levels in women with PCOS are significantly lower than in healthy controls (139–141). Consequently, PCOS women were treated with the daily administration of 220 mg zinc sulfate (containing 50 mg zinc) for 8 weeks and found that this regimen resulted in the improvement of their metabolic profiles (142). Similarly, the administration of 200 micrograms per day of selenium for 8 weeks in PCOS patients had beneficial effects on their insulin metabolism parameters, triglycerides and very low-density lipoprotein-C (VLDL-C) levels (143).

Combinations of AOXs are now increasingly being recommended for PCOS treatment. For example, a combination of NAC (1200 mg/day) and L-arginine (1600 mg/day) for 6 months to treat 8 oligo-menorrhoeic patients with PCOS helped restoring the menstrual function, as evidenced by the increased number of menstrual cycles, and significantly decreased the homeostatic model assessment (HOMA) index (144).

The potential for AOXs to replace metformin in the treatment of PCOS has also been explored. In a study by Oner and Muderris (2011), NAC was compared with metformin in 100 patients with PCOS (119). Both treatments resulted in significant reductions in BMI, hirsutism scores, fasting insulin levels, HOMA index, free testosterone levels, and menstrual irregularity compared to baseline values, demonstrating equal efficacy. Additionally, NAC significantly lowered both total cholesterol and LDL levels, while metformin only reduced total cholesterol (145).

Antioxidants were also used as adjuvant therapies in combination with ovulation induction agents in infertile patients with PCOS (146). In 2014, a RCT was conducted to evaluate the effectiveness of adding 3g of L-carnitine daily to a regimen of 250 mg clomiphene citrate, administered from day 3 to day 7 of the menstrual cycle, in anovulatory women with PCOS (147). The combination treatment significantly improved both ovulation rates and cumulative pregnancy rates compared to clomiphene citrate alone. Additionally, the L-carnitine group showed significant improvements in patient tolerability, lipid profiles, and BMI. Vitamin D is often used as a supplement in the treatment of PCOS and a meta-analysis showed that this may be beneficial for follicular development and menstrual cycle regulation in the PCOS patients (148). Chen et al. found that using vitamin E (100 mg/day during the follicular and luteal phases of the cycle) did not result in significant improvements in ovulation induction, clinical pregnancy, or ongoing pregnancy rates in infertile women with PCOS (149). In the latter study, a significantly lower dose of human menopausal gonadotropin (HMG) was required in these women, which is expected given that women with PCOS typically have high serum anti-mullerian hormone (AMH) levels and are more prone to developing ovarian hyperstimulation syndrome (OHSS).

In conclusion, while AOXs have shown promising potential in the treatment of PCOS, further research is essential to validate these findings. Additional studies are needed to determine which specific AOX or combination of AOXs offers the most benefit for managing this complex syndrome. New research can also help development of targeted therapies that can effectively address the underlying OS and other related factors in PCOS.

### Antioxidant therapy of endometriosis-associated infertility

Endometriosis affects about 10% of reproductive age women (150). Patients usually present with menstrual pain (dysmenorrhea) and/or infertility problems (12). OS has been implicated in the pathogenesis of endometriosis (12, 151–153). In these patients, OS results from excess ROS produced by the erythrocytes and apoptotic endometrial cells present in the endometriosis implants. ROS are also produced by the activated macrophages responsible for phagocytizing these apoptotic cells (11). Cells are damaged by OS-induced lipid peroxidation, which is proportional to the severity of the disease (154). It was also found that the peritoneal fluid of endometriosis patients contains high concentrations of various OS markers such as MDA, pro-inflammatory cytokines (interleukin-6 (IL-6), TNF-α, transforming growth factor- β (TGF-β) and IL-1β), angiogenic factors (IL-8 and VEGF), neural growth factor and oxidized LDL (ox-LDL) (155, 156). Besides their role in inflammation, these cytokines are detrimental to the processes of fertilization and embryo development: TNF-α increases the free implants adhesions and their invasiveness through its effect on matrix metallo-proteinases (157) while TGF-β hinders the local immune activity by inhibiting the natural killer cells which in turn impede the development of embryos (158). Further, mitochondria of ectopic endometrial stromal cells (ESCs) was found to generate excess ROS (159). A delicate redox balance between iron and heme oxygenase-1 (HO-1) has been identified, and this balance could be responsible for iron-induced OS in endometriotic cyst fluid (152). Additionally, a genetic role for OS in endometriosis has been proposed (22). A study investigated the genetic polymorphisms of four genes encoding AOX enzymes involved in OS and found that the variant genotypes for the CAT-262C, T polymorphism were significantly more frequent in individuals with endometriosis (160).

Consequently, AOXs have been utilized in the treatment of endometriosis, particularly for managing endometriosis-associated pain, with varying degrees of success (161–163). For instance, daily administration of a combination of vitamin E (1200 IU) and vitamin C (1000 mg) for eight weeks led to significant improvements in chronic pain, dysmenorrhea, and dyspareunia compared to placebo (164). Similarly, the severity of pelvic pain, dysmenorrhea and dyspareunia significantly decreased after 8 weeks of supplementation with vitamin C (1000 mg/day, 2 tablets of 500 mg each) and vitamin E (800 IU/day, 2 tablets of 400 IU each) compared to placebo (165). A recent study observed similar improvements following the administration of vitamin D (50,000 IU) every two weeks for 12 weeks in patients with endometriosis (166). Furthermore, a systematic review concluded that AOXs show promising results in treating endometriosis-related pain (167). However, the authors of the latter study emphasized the need for further studies to draw more definitive conclusions.

Few studies were conducted to evaluate AOX therapy in women with endometriosis-associated infertility (168, 169). Encouraging results were obtained following vitamin C supplementation in a group of women with endometriosis-associated infertility undergoing IVF treatment (170). In addition to human studies, animal research has also shown promise. For instance, studies conducted on rats demonstrated positive effects of AOX therapy on endometriosis-related infertility (146). However, these findings are preliminary and need to be confirmed through rigorous clinical trials in humans.

It is vital to take into account that AOXs should be given in proper dose and duration as excessive intake can lead to reductive stress (35). The delicate oxidant/AOX balance function is a double-edged blade. Although OS is implicated in cell death and apoptosis in endometriosis, upregulation of AOX functions in endometriotic cysts may result in restoration of cell survival (171, 172).

### Antioxidant therapy of premature ovarian failure and poor ovarian reserve

POF or premature menopause is the cessation of ovarian function before the age of 40 years. In POF, the ovaries lose their germinative and hormonal functions because of the exhaustion of the number of ovarian follicles before the typical age for physiological menopause which is around 50 years of age. Women with POF present with amenorrhea, hypergonadotropinism, and hypoestrogenism (173).

OS is increasingly recognized as a key factor influencing reproductive health, with effects that extend beyond fertility into broader physiological systems. Estrogen, owing to its intrinsic AOX properties, plays a critical role in mitigating OS. Its chemical structure enables the direct scavenging of free radicals while simultaneously enhancing the expression and activity of various AOX enzymes, thereby offering substantial protection against oxidative damage (174, 175). Elevated OS has been associated with systemic manifestations, including heightened lipid peroxidation, which correlates with mood disturbances, sleep disorders, and other health concerns (152). Dietary patterns enriched with AOX-rich components, such as the Mediterranean diet, have shown effectiveness in alleviating OS-related symptoms, underscoring the potential of dietary interventions in reducing oxidative damage (176). Similarly, hormone replacement therapy highlights the protective effects of estradiol in combating OS (177). These observations underscore the critical role of AOX-based strategies in advancing reproductive health and improving broader health outcomes, including those related to infertility.

OS has also been implicated in the process of ovarian ageing and an increased incidence of aneuploidy in ageing oocytes. In ageing oocytes, ROS attack the 8^th^ carbon atom of guanine leading to base mutations and mismatches in DNA replication (178). Accordingly, AOXs were explored as a method to delay this ageing process. Studies in mice have shown that peroxiredoxin-4 can be used to protect against ovarian ageing (179, 180). Similar results were reported with quercetin (181, 182). Ochiai et al. used resveratrol as an adjuvant therapy in women with poor ovarian reserve in an attempt to prevent premature menopause (183). Similarly, melatonin was used as an adjuvant for the prevention of POF during chemotherapy (184, 185), given its experimentally proven role in the female reproductive tract (186). Obviously, these promising reports need to be confirmed by prospective randomized trials.

### Antioxidant therapy of unexplained infertility

OS has been frequently associated with unexplained infertility. High levels of MDA, a marker of lipid peroxidation, were found in the peritoneal fluid of women with unexplained infertility, suggesting a potential role of OS in these cases (116). More recently, a study identified increased activity of prolidase, a type of matrix metalloprotease, in the plasma of women with unexplained infertility, proposing that this could contribute to implantation defects (187).

Given these findings, several attempts have been made to treat women with unexplained infertility using AOXs. Studies suggest that AOXs, such as CoQ10, vitamins C and E, melatonin, NAC, and alpha-lipoic acid (ALA), may improve oocyte quality, enhance endometrial receptivity, and restore hormonal balance by mitigating OS (188, 189). The evaluation often incorporates indirect markers such as improved fertilization rates, successful embryo development, and clinical pregnancy rates as proxies for oocyte quality. For example, CoQ10 has been shown to improve oocyte quality by enhancing mitochondrial function (190), while melatonin, may support both oocyte quality and endometrial receptivity (191).

Although some studies have reported positive outcomes when using AOX therapy in conjunction with other fertility treatments, the evidence remains mixed, and large-scale RCTs are needed to confirm its efficacy and safety. A study evaluated the role of adding NAC to clomiphene citrate in treating unexplained infertility but found the combination ineffective (192). Vitamin E supplementation in women with unexplained infertility undergoing controlled ovarian hyperstimulation and intrauterine insemination (IUI) was only effective in increasing the thickness of the endometrium without any effect on the pregnancy rate (193). In a more recent RCT, the addition of daily oral multivitamins and minerals to the stimulation regimen of patients with unexplained infertility treated with IVF/ICSI did not improve oocyte quality (metaphase II oocytes) or pregnancy rates in these women (194). These studies indicate the need for further research to evaluate the efficacy of AOX therapy in unexplained infertility and to determine whether OS is a causative factor or merely an associated finding in this condition.

### Antioxidant therapy of repeated miscarriages

RM or recurrent pregnancy loss is defined as the spontaneous loss of two or more pregnancies (195). It affects 1% of couples trying to conceive and in 50% of instances the cause is unknown (196). The relationship between OS and early pregnancy failure was first suggested by Jauniaux et al. (197). Subsequently, OS was explored as a cause of RM (198). Total oxidant level and OS index were significantly increased, and total antioxidant capacity, were significantly decreased in the serum of women with RM (199). Similarly, Ghneim et al. studied 25 women with RM and compared them to non-pregnant women and women with healthy pregnancies (200). They found significant reduction of SOD activities and serum levels of zinc, copper and manganese in healthy pregnant women compared to non-pregnant women with further reduction in RM patients. They suggested that dietary supplementation of Zn, Cu and Mn may have positive impact on these patients pre- and post-conception. The association between OS and RM was also supported by other studies (198, 201, 202). To the contrary, no statistically significant difference was found in the pre-gestational serum levels of OS indices in women with RM compared to controls (203).

Despite these conflicting data, AOXs were used in the treatment of RM with various claims of success. For example, a study compared the effects of administration of NAC 0.6 g + folic acid 500 μg/day in a group of 80 patients with a history of RM with an aged-matched group of 86 patients treated with folic acid 500 μ/day alone (204). They found a significantly increased rate of continuation of a living pregnancy and the take-home baby rate in the NAC supplementation group (P < 0.047, RR 1.98, 95% CI 1.3-4.0). However, a Cochrane review conducted in 2016 showed that AOXs did not help in preventing pregnancy loss (RR 1.12, 95% CI 0.24 to 5.29) but the authors suggested that more studies are needed to further explore this outcome (205).

## Antioxidant therapy and the outcome of medically assisted reproduction

OS in an important factor in the success of medically assisted reproduction (IUI, IVF and ICSI) (112, 128, 154). In these techniques, OS can result from endogenous as well as exogenous sources. These factors and the effect of AOXs on ART are discussed in this section (**Figure 1**).

### Impact of antioxidant therapy on intrauterine insemination outcomes

One of the main indications of IUI is the presence of oligozoospermia, asthenozoospermia or teratozoospermia or a combination of the three parameters in the male partner of the infertile couple. As mentioned previously, male partners with these defects exhibit OS in their semen (206–208). In addition, culture media used in the preparation of the semen as well as those used in cell cultures have varying amounts of ROS which adds to the OS burden to which spermatozoa are exposed (209, 210). Moreover, the process of sperm preparation deprives the spermatozoa from the natural AOXs present in the seminal plasma leading to accumulation of ROS. This is even more pronounced when centrifugation is used in the preparation (e.g. Percoll gradient separation). Cryopreservation and thawing are extra sources of OS when frozen semen is used for IUI (211).

Accordingly, AOXs are used to treat the male partners of the infertile couples as mentioned previously (16, 66). In addition, AOX supplementation of the sperm preparation medium for IUI was used in an attempt to counteract the effects of OS. Pentoxifylline exerts several AOX activities, such as the maintenance of GSH levels and mitochondrial viability (212). The addition of pentoxifylline to the sperm preparation medium resulted in improvement of semen characteristics in asthenozoospermic patients (213). Additionally, CPR was significantly increased by adding pentoxifylline to the semen preparation medium for IUI (27.5% versus 11.5% in the control group) (214).

Similarly, the addition of caffeine to the semen preparation medium for IUI was described (215). Finally, adjusting the sperm and embryo cryopreservation media by adding AOXs is used to mitigate the effect of OS (216).

### Impact of antioxidant therapy on IVF and ICSI outcomes

The techniques of IVF and ICSI exert a considerable amount of OS on the gametes and embryos. In addition to the endogenous and exogenous sources of OS mentioned previously to which spermatozoa are subjected, the oocytes and embryos are also subjected to similar OS (217). For example, oocytes lose their AOX protection contained in the cumulus cells during the denudation process. The changes of temperature and the osmotic and pH changes during the oocyte and embryo handling (denudation, pipetting, etc.) are all sources of OS (100). In addition, exogenous sources of OS to which the gametes and embryos are subjected include exposure to the atmospheric O_2_, the high oxygen concentration in the incubator (compared to the 2% concentration in the fallopian tube), volatile organic compounds present in the laboratory air or released from the consumables (plastic ware), visible light, humidity in the incubator and the type of mineral oil used. The processes of cryopreservation and thawing are also sources of OS (100). Consequently, many steps are taken in the IVF laboratory in order to counteract or diminish the effect of this OS (128).

Accordingly, men and women undergoing IVF or ICSI can be treated with AOXs in an attempt to improve the clinical results. Treatment of infertile men with the AOX “Menevit” for 3 months prior to IVF resulted in higher viable pregnancy rate (38.5%) compared to the control group (16%). The study enrolled 60 couples undergoing IVF-ICSI treatment, who were randomly assigned into two groups in a 2:1 ratio: one receiving the AOX Menevit and the other a placebo. Male participants were included based on evidence of OS, indicated by impaired sperm parameters such as poor morphology, reduced motility, or compromised membrane integrity, in conjunction with significant SDF (>25% TUNEL positivity). Female partners were excluded if they exhibited diminished ovarian reserve, defined as fewer than five oocytes retrieved in a prior IVF cycle or elevated early follicular phase FSH levels, or were aged over 39 years (218).​ The Cochrane review published in 2019 showed that although the use of AOXs for men prior to IVF did not improve the CPR (OR = 2.64, 95% CI 0.94 to 7.41), the LBR increased significantly (OR = 3.61, 95% CI 1.27). However, the authors of the review noted that these data were based on 2 RCTs only and that further studies are needed (11).

Women undergoing IVF and ICSI were also treated with AOXs prior to the procedure with variable claims of success. These treatments include NAC, melatonin, coQ10, thiamine, riboflavin, niacin B3, vitamins B6 and B12, folate, vitamins A, C, D and K, calcium, phosphorus, magnesium, sodium, potassium, chloride, iron, zinc, copper, selenium, iodine, vitamin E, vitamin K, L-arginine, inositol, biotin, pantothenic acid, eicosatetraenoic acid (EPA), docosahexaenoic acid (DHA) and various combinations of the above (188). The treatment is usually given for 3 to 6 months prior to the procedure. Pre-treatment of IVF patients with AOXs improved embryo quality, particularly in poor responders (219). Indeed, a significantly lower glutathione-S-transferase (GST) enzyme activity and significantly high MDA levels have been shown in the cumulus cells of poor responders compared to high responders (220). However, the Cochrane review conducted by Showell et al. in 2020 showed no improvement in the CPR (OR = 1.15, 95% CI = 0.95 – 140) or LBR (0.94, 95% CI = 0.41- 2.15) when AOXs were given to women treated with IVF or ICSI (188). In this Cochrane review, the LBR was calculated from 2 small RCTs and it is therefore clear that more studies are needed, particularly in view of the multifactorial nature of IVF and ICSI.

Finally, supplementation of embryo culture media with AOXs was shown to improve the quality of the embryos, the implantation rate as well as the CPR in IVF/ICSI cycles (221, 222). Furthermore, the addition of a combination of AOXs (ALC, NAC and α-lipoic acid) to the cryoprotectant medium of mice embryos resulted in increased blastocyst cryo-survival and viability post-vitrification (216).

The outcome of ICSI is known to be influenced by a myriad of factors. In a recent Cochrane review, it was stated that the chance of live birth with ICSI is between 30% and 41% (223). Consequently, efforts are directed to maximize ICSI outcomes. Increased OS has been identified as an important factor governing success of ICSI (112). Thus, it is plausible to use AOX in an endeavor to optimize the outcome of ICSI. Although the list of publications on the use of AOX in couples undergoing ICSI is steadily growing, knowledge on the relationship between AOX and ICSI outcomes is lacking (224). An RCT including 85 women with different indications for ICSI who have been supplemented with AOX daily from the cycle preceding ICSI cycle, versus 85 women who were not given any AOX acting as controls, concluded that AOX therapy did not change clinical or laboratory outcomes (225). However, A recent systematic review indicated a favorable impact of AOX on pregnancy outcomes following ICSI (224). In a recent systematic review and meta-analysis of RCTs, it was concluded that oral supplementation of CoQ10 may increase clinical pregnancy rates, without an effect on LBR, and MR compared with placebo or no-treatment in women with infertility undergoing ICSI (226). Moreover, a recent meta-analysis concluded that melatonin treatment significantly increases the CPR but not LBR in ART cycles. Melatonin treatment also increased the number of oocytes collected, maturate oocytes, and good quality embryos. There was no evidence suggesting that melatonin treatment increases the adverse events in ART cycles. However, the findings of this meta-analysis should be explained cautiously due to remarkable heterogeneity of the included IVF patients (227). AOXs such as inositol, L-carnitine, CoQ10, and ALC may be helpful for women undergoing ICSI or in cases of previous ICSI failure (226). However, the role of AOXs in increasing LBR is limited by low-quality evidence (188). Indeed, conducting large controlled clinical trials using AOXs supplementation is mandatory to evaluate their influence on ICSI outcome.

## Future directions

Available research on AOX therapy is surrounded by several limitations and biases, including small sample sizes, heterogenous patient populations and inclusion criteria, poor study designs, and inconsistent reporting of clinical outcomes. Additionally, the use of a wide range of AOXs (either single or in combinations) and the lack of standardized dosages in different studies preclude the ability to draw a firm conclusion on the efficacy of AOX supplementation in solving infertility problem. This has led to the skepticism of the current guidelines by professional societies regarding AOX therapy for infertility. This may also explain the considerable variability that currently exists in practitioners’ attitude towards the prescription of AOX therapy for the purpose of overcoming infertility issues. Such variability surrounds many aspects of AOX therapy including pre-testing the patient for OS, the choice of a specific AOX or AOX combination, proper dose, optimum duration of therapy and the desired outcome. Therefore, there is a great demand for high-quality research to clarify the exact role of AOXs in the context of infertility treatment.

Future studies should focus on careful diagnostic work-up to assess OS indices as an essential pre-request before starting AOX therapy (206). Such test would be helpful for selecting patients with high levels of OS who are candidates for AOX therapy, and for monitoring the treatment response. This in turn requires the availability of reliable tools for confirming the diagnosis of OS and evaluation its severity. Indeed, this is technically difficult in women. However, assessment of seminal OS in men is feasible using some tools such as the MiOXSYS System that can help assess oxidation-reduction potential (ORP) levels in semen and seminal plasma (207). The test would help personalize treatment for cases with high seminal ORP such as idiopathic infertility, varicocele or those with genital tract infection. The 6th Edition of the World Health Organization (WHO) manual for human semen analysis classifies seminal OS testing as a research tool, emphasizing the lack of robust evidence to confirm its clinical utility (208). Despite this designation, OS testing is extensively utilized in andrology research, clinical laboratories, and ART centers, underscoring its recognized importance in advancing the understanding of male fertility.

Future studies are also warranted to investigate the impact of AOX on LBR, MR, and SDF since only few well-designed studies have analyzed these outcomes in infertile patients following AOX therapy. Additionally, future studies are needed to determine the proper treatment duration, optimum dosage as well as the ideal supplement (single vs. combined AOX) (206). Furthermore, it is essential to ensure that there are no negative consequences of utilizing AOXs as a therapeutic option for infertile men.

## Conclusion and take-home message

OS plays a significant role in couples’ infertility. The theoretical basis for AOX therapy to counteract the harmful effects of ROS on fertility is feasible. In infertile men, it is hypothesized that cases with elevated seminal OS may benefit from AOX therapy. Hence, several AOXs, including vitamins E and C, carotenoids, carnitine, cysteine, CoQ10, selenium, zinc, and folate, have been employed as potential therapeutic interventions for male infertility. However, results of clinical trials remain controversial, and current guidelines from major professional societies offer no definitive recommendations regarding AOX use in male infertility.

AOXs have shown promise in the management of PCOS, endometriosis, poor ovarian reserve and unexplained infertility. Also, AOX supplements such as inositol, L-carnitine, ALC and CoQ10 have shown potential for women undergoing ICSI, particularly those with previous ICSI failure. However, current evidence supporting AOX in improving important reproductive outcomes such as LBR remains low, necessitating further studies. Additionally, further research is needed to evaluate the exact role of AOX therapy and to identify the most effective single AOX or AOX combinations in these conditions.
